# Parasitose intestinale simulant une appendicite à l’échographie: à propos de deux cas

**DOI:** 10.11604/pamj.2015.21.322.3339

**Published:** 2015-08-31

**Authors:** Andrianah Emmylou Prisca Gabrielle, Rajaonarison NyOny Narindra Lova Hasina, RandrianantenainaFarlahy Ravelonarivo, Ahmad Ahmad

**Affiliations:** 1 Département d'Imagerie Médicale et Radiodiagnostic, Centre Hospitalier Universitaire Joseph Andrianavalona Ampefiloha, Antananarivo, Madagascar

**Keywords:** Parasitose intestinale, appendicite, parasitoses intestinales, Intestinal parasites, appendicitis, intestinal parasitosis

## Abstract

L'objectif de ce travail est d'apporter un diagnostic différentiel d'appendicite à l’échographie sur la région de la fosse iliaque droite, destiné notamment aux échographistes mal entrainés ou débutant. Nous rapportons deux cas de parasitoses intestinales découvertes au niveau de la fosse iliaque droite simulant une appendicite. Le premier cas concernait une femme de 45 ans, sans antécédent particulier, référée pour échographie abdominale suite à une douleur de la fosse iliaque droite, apyrétique, sans trouble de transit. L'examen échographique par voie sus-pubienne de la fosse iliaque droite (FID) objectivait une structure arrondie à paroi épaisse, de 8 mm de diamètre en coupe transversale et tubulée à extrémité borgne en coupe longitudinale, à contenu hétérogène hypoéchogène en coupe longitudinale et une sensibilité de cette zone au passage de la sonde. La biologie présentait une hyperéosinophilie et à l'examen des selles par recherche de kyste, amibe et oeufs parasitaires, on décelait des oeufs parasitaires. Le deuxième cas était un garçon de 15 ans, sans antécédent particulier. Son échographie était indiquée suite à une douleur abdominale aigue fébrile. Les images échographiques sur la FID montraient une structure arrondie de 7 mm de diamètre et à paroi épaisse hyperéchogène en coupe transversale et en coupe longitudinale, elle était tubulée borgne, immobile et sensible au passage de la sonde. La numération formule sanguine rapportait une hyperleucocytose à prédominance polynucléaire. Au total, la parasitose intestinale peut mimer un aspect échographique d'une appendicite, que tout échographiste notamment les débutants et peu expérimentésdoit éliminer.

## Introduction

L'appendicite aigue est une pathologie fréquente et engendre une urgence chirurgicale. L’échographie reste l'examen de première intention chez l'enfant et les sujets jeunes [1]. La définition échographique d'une appendicite est portée sur son diamètre, sa paroi, son contenu et la sensibilité au passage du transducteur [2]. La parasitose intestinale est une maladie par transmission féco-oral. Elle touche plus d'un millions de personnes, avec un pic dans l'enfance [3] et banalisée parfois. Sa gravité réside sur l'avènement d'une occlusion intestinale. L’échographie permet de détecter les parasites intestinales et se caractérisent par une image allongée tubulée, en rail en coupe longitudinale et de diamètre variable [4]. L’échographie permet d'apporter le diagnostic d'appendicite, mais surtout de rechercher les complications et d’écarter les diagnostics différentiels. Ainsi, l'objectif de ce travail est d'apporter un diagnostic différentiel de l'appendicite aigue, sur la fosse iliaque droite à l’échographie, surtout pour les échographistes mal entraînés et débutants.

## Patient et observation

### 1^er^ cas

Il s'agissait d'une femme âgée de 46 ans, sans antécédent particulier, non ménopausée, ayant trois grossesses, trois parités, sans avortement. Son dernier déparasitage daté de son enfance. En effet, cette patiente présentait une douleur abdominale diffuse et prédominante sur la fosse iliaque droite, survenue de façon brutale, à type de torsion, intense avec une échelle visuelle analogique (EVA) de 8/10, sans facteurs déclenchants ni des troubles du transit et évoluait dans un contexte apyrétique. La patiente était en bon état général, sa température prise le matin était de 37. Lesexamens physiques révélaient un syndrome douloureux de la FID, et les autres étaient sans anomalie particulière, notamment la sphère gynécologique.

**Figure 1:**
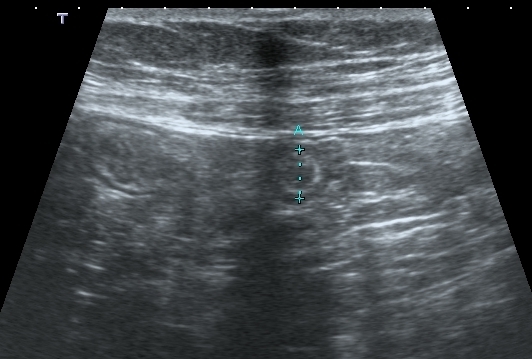
Coupe transversale échographique, d'une structure arrondie bien limitée, de 8 mm de diamètre, à contenu hétérogène hyperéchogène, sur la région de la fosse iliaque droite (Parasitose intestinale)

**Figure 2:**
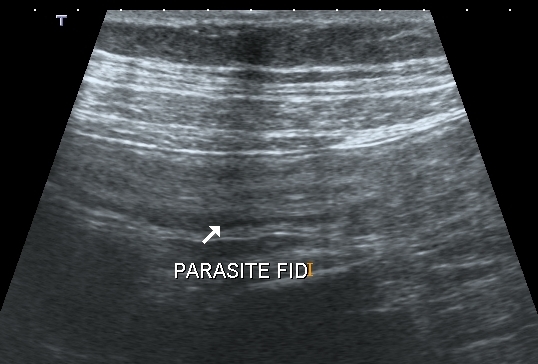
Coupe longitudinale échographique, formation tubulée, en ligne parallèle, à extrémité borgne, à paroi épaisse de 4 mm, à contenu hétérogène, immobile à la compression, sur la fosse iliaque droite (Parasitose intestinale)

Des examens biologiques, comme la numération de la formule sanguine et des selles ont révélé une hyperéosinophilie et la présence d'oeufs d'ascaris dans les selles. L’échographie abdominale par voie sus-pubienne avec utilisation d'une sonde de haute fréquence de 7,5 MHz effectuait sur la région de la FID a objectivé en coupe transversale, une structure arrondie, bien limitée, de 8 mm de diamètre environ, à contenu hétérogène hyperéchogène et en coupe longitudinale cette structure était tubulée avec une extrémité borgne, à paroi hyperéchogène épaisse, et apéristaltique. On notait une sensibilité de la FID lors du passage de la sonde(Figure 1, Figure 2). Ces images posaient ainsi le problème de diagnostic d'appendicite et de parasitose. Finalement, la patiente a été traité avec un anti-parasitaire, où elle affichait une bonne évolution, marquée par la disparition des douleurs et des oeufs de parasites dans les selles.

### 2^ème^ cas

Il s'agissait d'un garçon de 15 ans, sans antécédent particulier et prenait ses anti-parasitaires irrégulièrement. Il présentait une douleur abdominale de survenue aigue prédominant à la FID, à type de piqûre, intense avec une EVA à 9/10, associée à des vomissements alimentaires, sans trouble du transit ni facteurs déclenchant. Des toux sèches étaient un autre signe associé. Le patient était fébrile, avec une température de 38,7° le matin. Les examens physiques rapportaient un assez bon état général et un syndrome appendiculaire aigu. On notait un syndrome de condensation pulmonaire basale gauche. La biologie révélait une hyperleucocytose à prédominance polynucléaire et la présence d'oeufs de parasite dans les selles.

L’échographie abdominale par utilisation de sonde superficielle, montrait en coupe transversale une image arrondie, aux contours réguliers, de 7 mm d’épaisseur, à paroi épaisse, hétérogène hypoéchogène et à contenu hétérogène hyperéchogène en coupe longitudinale elle se présentait sous une forme allongée, à extrémité borgne, immobile, et sensible à la compression (Figure 1). Ces images faisaient un souci de diagnostic entre une appendicite et une parasitose intestinale. Finalement, le patient a été traité comme une pneumopathie bactérienne et une parasitose intestinale.

## Discussion

L'appendicite est une pathologie fréquemment rencontrée en milieu chirurgical et représente les causes prédominantes des douleurs de la FID [5, 6]. Le taux de détection par échographie d'un appendice normal varie selon le mode, dont le mode harmonique, qui pour certains auteurs [7, 8] représentait 49,2% de son étude chez les sujets asymptomatiques contre 45,9% pour Simornasky par utilisation d'une échographie conventionnelle [9]. L'appendice normal était décrit à l’échographie par des auteurs [2, 10], à partir de son diamètre inférieur à 6 mm, et de l’épaisseur de la paroi qui était éest paissi, inférieur à 3 mm quibien dédifférenciée. La présence de gaz intra-luminal était un des critères de normalité rapporté par Poljak et al [11].

Plusieurs auteurs décrivaient une haute sensibilité et une forte spécificité de l’échographie dans le diagnostic de l'appendicite [12], mais sa limite est opérateur-dépendant [13]. Les aspects échographiques de l'appendicite étaient décrits par Kessler N et al par une dilatation appendiculaire, dont le diamètre est au-delà de 6 mm qui est un signe spécifique, associée à une paroi épaisse, qui perd sa dédifférenciation [2, 10]. Franke C et al rapporte la présence de stercolithe dans la lumière appendiculaire [13].

Pour nos cas, les images échographiques découvertes au niveau de la fosse iliaque droite en coupe transversale étaitarrondies et avient un diamètre de 8 mm pour le premier cas et 7 mm, avec une paroi épaisse supérieure à 3 mm, sans dédifférenciation, immobile et sensible à la compression. Ces aspects échographiques pourraient correspondre à une appendicite. La parasitose intestinale est une maladie des mains sales [3]. L’échographie détecte également les parasitoses intestinales dont l’*ascaris lombricoïdes* qui fait partie des helminthiases intestinales qui affectent le plus de personnes [14]. Cette technique permet de détecter rapidement les parasitoses intestinales [15]. La description échographique de l'ascaris est rapportée comme des lignes parallèles et en rail pour son contenu, leur diamètre est variable, ils sont mobiles si sont vivants [4]. Et dans notre travail, les coupes échographiques de nos cas monétraient des images tubulées, avec des parois traçant des lignes parallèles à contenu hétérogène, dessinant des images en rail, pouvant correspondre à un parasite intestinal mort.

Une confrontation des images échographiques avec les données clinico-biologiques ont pu éliminer une appendicite et à retenir une parasitose intestinale.

## Conclusion

L’échographie abdominale tient une place importante dans le diagnostic d'une appendicite et permet la recherche de parasitose intestinale. L'helminthiase intestinale peut prêter un diagnostic d'appendicite, qu'il faut penser à éliminer parmi les autres diagnostics différentiels de cette pathologie inflammatoire au niveau de la fosse iliaque droite, surtout pour les échographistes peu expérimentés ou débutants. Toutefois une confrontation des données clinico-biologiques et échographiques est souhaitable.

## References

[1] Mathias J, Bruot O, Ganne P-A, Laurent V, Regent D (2008). Appendicite.

[2] Kessler N, Cyteval C, Gallix B (2004). Appendicitis: evaluation of sensitivity, specificity, and predictive values of US, Doppler US, and laboratory findings. Radiology..

[3] Ozmen MN, Oguzkurt L, Ahmet B, Akata D, Akhan O (1995). Ultrasonographic diagnosis of intestinal ascariasis. Pediatr Radiol..

[4] Agostini S (1993). Manuel d'ultrasonologie générale de l'adulte.

[5] Reginelli A, Mandato Y, Solazzo A, Berritto D, Iacobellis F, Grassi R (2012). Errors in the radiological evaluation of the alimentary tract: part II. Semin Ultrasound CT MR..

[6] Macarini L, Stoppino LP, Centola A, Muscarella S, Fortunato F, Coppolino F, Della Valle N, Ierardi V, Milillo P, Vinci R (2013). Assessment of activity of Crohn's disease of the ileum and large bowel: proposal for a new multiparameter MR enterography score. Radiol Med..

[7] Yucel C, Ozdemir H, Asik E, Oner Y, Isik S (2003). Benefits of tissue harmonic imaging in the evaluation of abdominal and pelvic lesions. Abdom Imaging..

[8] Shapiro RS, Wagreich J, Parsons RB, Stancato-Pasik A, Yeh HC, Lao R (1998). Tissue harmonic imaging sonography: evaluation of image quality compared with conventionalsonography. AJR Am J Roentgenol..

[9] Simonovsky V (1999). Sonographic detection of normal and abnormal appendix. Clin Radiol..

[10] Rioux M (1992). Sonographic detection of the normal and abnormal appendix. AJR Am J Roentgenol..

[11] Poljak A, Jeffrey RB, Kernberg ME (1991). The gas-containing appendix: potential sonographicpitfall in the diagnosis of acute appendicitis. J Ultrasound Med..

[12] Rettenbacher (2001). Outer Diameter of the Vermiform Appendix as a Sign of Acute Appendicitis: Evaluation at US. Radiology.

[13] Franke C (1999). Ultrasonography for Diagnosis of Acute Appendicitis: Results of a Prospective Multicenter Trial. World J Surg Société Internationale de Chirurgie..

[14] Park MS, Kim KW, Ha HK, Lee DH (2008). Intestinal parasitic infection. Abdom Imaging..

[15] Tariq M, Naheed M, Saeed Q, Muhammad I, Sarwat (2001). Ultrasonographic Appearance of Ascarislumbricoidesin the Small Bowel. J Ultrasound Med..

